# Sensitization and Deceased-Donor Kidney Transplant Access in France

**DOI:** 10.1016/j.ekir.2026.106340

**Published:** 2026-02-01

**Authors:** Dominique Bertrand, Philippe Gatault, Coralie Poulain, Charlotte Colosio, Clément Danthu, Agnès Duveau, Laure Ecotière, Cyril Garrouste, Yannick Le Meur, Marie-Pascale Morin, Valérie Châtelet, Sophie Caillard, Dany Anglicheau

**Affiliations:** 1 Department of Nephrology, Kidney Transplantation and Hemodialysis, Rouen University Hospital, Rouen, France; 2 Department of Nephrology, Kidney Transplantation and Hemodialysis, Tours, University Hospital, Tours, France; 3 Department of Nephrology, Kidney Transplantation and Hemodialysis, Amiens University Hospital, Amiens, France; 4 Department of Nephrology, Kidney Transplantation and Hemodialysis, Reims University Hospital, Reims, France; 5 Department of Nephrology, Kidney Transplantation and Hemodialysis, Limoges University Hospital, Limoges, France; 6 Department of Nephrology, Kidney Transplantation and Hemodialysis, Angers University Hospital, Angers, France; 7 Department of Nephrology, Kidney Transplantation and Hemodialysis, Poitiers University Hospital, Poitiers, France; 8 Department of Nephrology, Kidney Transplantation and Hemodialysis, Clermont Ferrand University Hospital, Clermont Ferrand, France; 9 Department of Nephrology, Kidney Transplantation and Hemodialysis, Brest University Hospital, Brest, France; 10 Department of Nephrology, Kidney Transplantation and Hemodialysis, Rennes University Hospital, Rennes, France; 11 Department of Nephrology, Kidney Transplantation and Hemodialysis, Caen University Hospital, Caen, France; 12 Department of Nephrology, Kidney Transplantation and Hemodialysis, Strasbourg University Hospital, Strasbourg, France; 13 Department of Nephrology and Kidney Transplantation, Necker Enfants Malades Hospital, Assistance Publique-Hôpitaux de Paris, Paris, France

**Keywords:** allocation policy, desensitization, Fine and Gray model, HLA sensitization, kidney transplantation

## Abstract

**Introduction:**

Highly sensitized kidney transplant candidates have reduced access to deceased-donor kidney transplantation (DDKT) and worse waitlist outcomes. In France, allocation relies on sensitization-based prioritization through the acceptable-antigen pathway driven by the *Taux de Greffons Incompatibles* (TGI)’ however, the sensitization threshold at which this system fails to preserve equity remains unclear.

**Methods:**

We conducted a retrospective multicenter cohort study including 14,485 adult kidney transplant candidates listed between 2011 and 2021. Sensitization was measured using TGI (0–100) and reported using calculated panel reactive antibody (cPRA)-labeled strata for international readability. Access to DDKT was the primary outcome, with living-donor transplantation, death, and delisting treated as competing events. The secondary outcome was waitlist attrition (death or delisting). Fine–Gray models adjusted for key demographic, clinical, and immunologic variables were truncated at 10 years.

**Results:**

Access to DDKT declined progressively with increasing sensitization and fell sharply from cPRA ≥ 96%. Compared with cPRA 0%, the adjusted subdistribution hazard ratio (SHR) for DDKT was 0.781 at 96%, 0.658 at 97%, 0.701 at 98%, 0.353 at 99%, and 0.082 at 100%. Conversely, the risk of waitlist attrition increased from cPRA ≥ 97% (sHR: 1.793), reaching 2.713 at 100%. Candidates with cPRA of 85% to 95% retained preserved access, consistent with the intended effect of the acceptable-antigen pathway.

**Conclusion:**

In the French TGI-based allocation system, cPRA ≥ 96% marks a threshold of impaired access to DDKT, whereas cPRA ≥ 97% identifies excess waitlist attrition. These findings define an ultrasensitized subgroup insufficiently served by current prioritization and support refined allocation strategies within the 96% to 100% range.

## Graphical abstract

**Figure:**
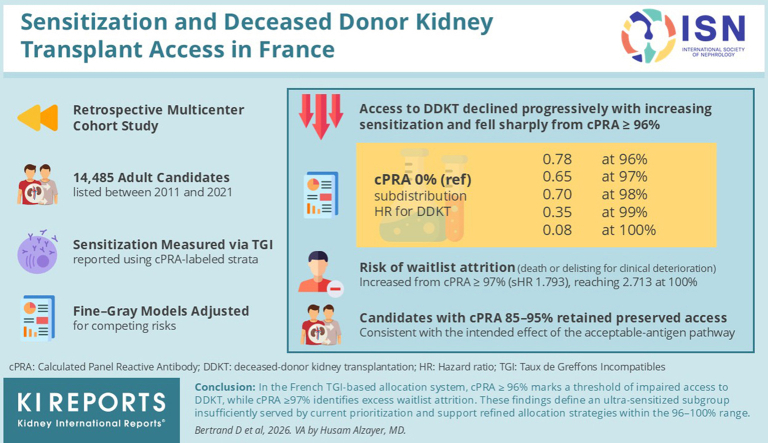

Kidney transplantation is the optimal treatment for patients with end-stage renal disease, offering a substantial survival advantage and improved quality of life compared with dialysis.^1^, ^2^, ^3^ However, access to transplantation remains constrained by the chronic imbalance between organ demand and supply.^4^ Prolonged waiting times are associated with increased morbidity and mortality, particularly among immunologically complex candidates.^5^

Highly sensitized patients—those with circulating anti–human leukocyte antigen (HLA) antibodies from transfusions, pregnancies, or prior transplants—face the greatest barriers.^6^^,^^7^ Antibody specificities restrict donor compatibility, prolong waiting, and raise the risk of death or delisting.^5^ To mitigate this, allocation policies have introduced prioritization for sensitized candidates. In the United States, the Kidney Allocation System awards points by cPRA, improving access at very high cPRA (≥ 98%).^8^ In Eurotransplant, the Acceptable Mismatch (AM) program identifies nonreactive antigens to expand compatible donor profiles.^9^ Despite these advances, access for the most highly sensitized (e.g., cPRA ≥ 99%–100%) remains limited and desensitization is variably applied.^10^^,^^11^

Sensitization is generally quantified by cPRA, which estimates the share of donors against whom a candidate has clinically relevant anti-HLA antibodies.^12^ Reference panels and recalculation policies differ across systems (e.g., US policy updates vs. fixed Eurotransplant panels).^8^^,^^13^ In France, sensitization is measured with the TGI, created by the Agence de la Biomédecine. The TGI is an integer percentage (0%–100%) of ABO-identical deceased donors procured nationally over the previous 5 years against whom a candidate has ≥ 1 clinically relevant unacceptable anti-HLA specificity (HLA-A, -B, -DR, -DQ) recorded in the French National Transplant Registry (CRISTAL); it is computed without considering the number of HLA mismatches and recalculated nightly, providing a dynamic measure of incompatibility.^14^ Although conceptually similar to cPRA in that it expresses a percentage estimate of donor incompatibility, TGI differs from US system or Eurotransplant effectively in its underlying reference population and calculation method; therefore, the 2 terms should not be considered interchangeable. For international readability, we report results using cPRA-labeled strata with numerically identical integer values, while the underlying sensitization metric remains TGI. The TGI underpins eligibility and ranking within the French AM program (threshold ≥ 85%). However, its real-world impact on access across the full sensitization spectrum has not been systematically evaluated, particularly for very high TGI levels (≥ 95%–97%), where it is unclear whether current prioritization overcomes extreme incompatibility.

To address this gap, we conducted a large, multicenter retrospective cohort study across 13 French transplant centers (SPIESSER group). Our primary objective was to assess access to DDKT by sensitization level (reported in internationally comparable cPRA categories). Secondary objectives were to evaluate death or delisting across cPRA strata and to identify operational sensitization thresholds associated with significantly impaired access, with the aim of informing allocation policy and selection for desensitization.

## Methods

### Study Design and Population

We conducted a retrospective, multicenter cohort study including all adult patients listed for kidney transplantation across 13 French transplant centers participating in the SPIESSER group. All patients without temporary or permanent contraindications to transplantation were eligible for inclusion. The study period extended from January 1, 2011, to December 31, 2021, with follow-up through January 1, 2023, and truncated at 10 years for the primary analyses.

Patients were identified from the CRISTAL nationwide comprehensive registry maintained by the Agence de la Biomédecine (the national authority overseeing transplantation and allocation in France) and anonymized before analysis. The following variables were collected at listing: demographic characteristics, dialysis status, number of previous transplantations, blood group, renal disease, and immunological sensitization parameters. Although basic demographic characteristics were available, detailed comorbidity data were not consistently recorded in a standardized manner across participating centers and were therefore not included in the multivariable analyses.

In accordance with French legislation (Loi Jardé), ethics committee approval was not required for this noninterventional retrospective study using anonymized data. The study adhered to the principles of the Declaration of Helsinki and the Declaration of Istanbul on Organ Trafficking and Transplant Tourism.

### Exposure Variable: Sensitization Level

The primary exposure variable was sensitization level, measured in France using the TGI. The TGI is an integer-based percentage (0–100%) that quantifies the proportion of ABO-identical deceased donors procured in France over the previous 5 years against whom a candidate is immunologically incompatible because of clinically relevant unacceptable anti-HLA specificities (HLA-A, -B, -DR, and -DQ) recorded in the national allocation database (CRISTAL). The calculation does not take into account the number of HLA mismatches and is recalculated nightly, providing a dynamic measure of donor incompatibility.

Clinically relevant unacceptable anti-HLA specificities are defined based on anti-HLA antibodies identified by single-antigen bead assays and validated by local immunology laboratories according to national guidelines before entry into CRISTAL.

For international readability, sensitization strata are reported using cPRA-labeled categories with numerically identical integer values, while the underlying sensitization metric remains TGI. Equivalence with US or Eurotransplant cPRA reference populations should not be assumed.

For the main analysis, cPRA was categorized into the following strata: 0%; 1% to 25%; 26% to 50%; 51% to 84%; 85% to 89%; 90% to 94%; 95%; 96%; 97%; 98%; 99%; 100%. This categorization allowed both an evaluation of intermediate sensitization levels and a focused analysis of the highest cPRA ranges, where immunological barriers are most pronounced.

### Outcomes

The primary outcome was access to DDKT during follow-up. Living-donor kidney transplantation (LDKT), death while on the waiting list, and permanent removal from the list due to clinical deterioration were treated as competing events. The secondary outcome was the composite of death or delisting for medical deterioration, with any transplantation (deceased or living donor) treated as the competing event.

### Statistical Analysis

Descriptive statistics were used to summarize baseline characteristics. Continuous variables were expressed as mean ± SD or median (interquartile range), and categorical variables as counts and percentages.

Cumulative incidence functions were estimated for each cPRA category using the Fine and Gray method for competing risks. SHRs and 95% confidence intervals were derived from multivariable Fine and Gray regression models.

A single competing-risk model was constructed for each outcome of interest. For the primary analysis assessing access to DDKT, competing events were death, delisting for medical deterioration, and LDKT. For the secondary analysis assessing waitlist attrition, transplantation was treated as the competing event.

Covariates included in both models were as follows: age at listing (continuous), gender (reference: female), dialysis status at listing (yes/no; reference: no), ABO blood group (reference: A), cPRA category (reference: 0%), number of previous kidney transplantations (categorical; reference: 0), and primary renal disease (categorical; reference: chronic tubulointerstitial kidney disease / uropathy). LDKT was not included as a covariate, because it represents a postbaseline event and was handled as a competing risk.

All primary analyses were limited to a 10-year follow-up period to improve clinical interpretability of waitlist outcomes.

Finally, the proportional hazards assumption was assessed using a cause-specific Cox model and Schoenfeld residuals. This diagnostic step was performed for descriptive purposes only, because the Fine and Gray method is not constrained by this assumption.

All analyses were conducted using R software (version 4.3.3, 2024-02-29), with the cmprsk, and survival packages. A 2-sided *P*-value < 0.05 was considered statistically significant.

## Results

### Study Population

Between 2011 and 2021, 14,485 adult candidates were actively listed for kidney transplantation across 13 French centers. The mean age at listing was 54.4 ± 14.4 years, 83.1% were on dialysis, and 63.7% were male. ABO distribution was 42.0% group A, 4.3% group AB, 11.2% group B, and 42.5% group O.

Patients were stratified into 4 cPRA groups: 0% (*n* = 7937), 1% to 84% (*n* = 4685), 85% to 95% (*n* = 803), and 96% to 100% (*n* = 1060) (Table 1). Age decreased and the proportion of women increased with higher sensitization. Dialysis prevalence increased from 80.8% to 93.7% across cPRA strata. Glomerular disease was the most common etiology (Table 2), whereas diabetic nephropathy was more frequent in unsensitized candidates. Previous kidney transplants were strongly associated with higher cPRA as follows: from 4.7% at cPRA 0% to 66.5% at ≥ 96%, including 15.9% with ≥ 2 prior grafts.

**Table 1:** Baseline clinical and immunological characteristics of the study population

Characteristics	Overall (*N* = 14485)	cPRA = 0% (*n* = 7937)	cPRA = 1%–84% (*n* = 4685)	cPRA = 85%–95% (*n* = 803)	cPRA = 96%–100% (*n* = 1060)
Age, yr, mean ± SD	54.44 ± 14.36	55.52 ± 14.62	53.89 ± 14.13	52.02 ± 13.29	50.66 ± 13.20
Dialysis at registration, *n* (%)	12033 (83.1)	6416 (80.8)	3912 (83.5)	712 (88.7)	993 (93.7)
Gender: Female, *n* (%)	5254 (36.3)	2203 (27.8)	1984 (42.3)	475 (59.2)	592 (55.8)
Blood group: A, *n* (%)	6084 (42)	3488 (43.9)	1828 (39)	327 (40.7)	441 (41.6)
Blood group: AB, *n* (%)	617 (4.3)	354 (4.5)	167 (3.6)	39 (4.9)	57 (5.4)
Blood group: B, *n* (%)	1629 (11.2)	821 (10.3)	604 (12.9)	88 (11)	116 (10.9)
Blood group: O, *n* (%)	6155 (42.5)	3274 (41.2)	2086 (44.5)	349 (43.5)	446 (42.1)
History of previous kidney transplantation, *n* (%)					
1	1908 (13.2%)	352 (4.4%)	662 (14.1%)	358 (44.6%)	705 (66.5%)
> 1	329 (2.3%)	22 (0.3%)	77 (1.6%)	61 (7.6%)	169 (15.9%)
KT, *n* (%)	10637 (73.4)	6155 (77.5)	3319 (70.8)	628 (78.2)	535 (50.5)
Living donor, *n* (%)	1509 (10.4)	881 (11.1)	529 (11.3)	37 (4.6)	62 (5.8)
Mean wait before KT± SD (yr)	1.85 ± 1.66	1.59 ± 1.48	2.13 ± 1.79	2.11 ± 1.70	2.71 ± 2.15
Death, *n* (%)	1155 (8.0)	581 (7.3)	370 (7.9)	46 (5.7)	158 (14.9)
Removal for clinical deterioration. *n* (%)	642 (4.4)	319 (4.0)	207 (4.4)	37 (4.6)	79 (7.4)
Still waiting at the end of the follow-up, *n* (%)	2051 (14.2)	882 (11.1)	789 (16.8)	92 (11.5)	288 (27.2)
Mean wait ± SD (yr)	3.71 ± 2.14	3.24 ± 1.65	3.56 ± 1.90	4.95 ± 2.73	5.14 ± 2.97

cPRA, calculated panel reactive antibody; KT, kidney transplantation.

This table presents the baseline demographic, clinical, and immunological characteristics of the patients included in the study. Continuous variables are expressed as mean ± SD or median [interquartile range] as appropriate. Categorical variables are presented as number (percentage). Data are stratified according to the cPRA status.

**Table 2:** Distribution of primary kidney disease by cPRA category at listing

Primary kidney disease	cPRA = 0% (*n* = 7937)	cPRA = 1%–84% (*n* = 4685)	cPRA = 85%–95% (*n* = 803)	cPRA = 96%–100% (*n* = 1060)
Diabetic nephropathy	995 (12.5%)	549 (11.7%)	53 (6.6%)	88 (8.3%)
Vascular nephropathy	814 (10.3%)	467 (10.0%)	51 (6.4%)	66 (6.2%)
Glomerular disease	1968 (24.8%)	1064 (22.7%)	237 (29.5%)	344 (32.5%)
Chronic tubulointerstitial nephropathy / uropathy	1005 (12.7%)	649 (13.9%)	134 (16.7%)	196 (18.5%)
Hereditary kidney disease	1451 (18.3%)	940 (20.1%)	160 (19.9%)	155 (14.6%)
Unknown or undetermined nephropathy	1309 (16.5%)	793 (16.9%)	124 (15.4%)	153 (14.4%)
Other	395 (5.0%)	223 (4.8%)	44 (5.5%)	58 (5.5%)

cPRA, calculated panel reactive antibody.

Primary renal diagnoses are stratified according to cPRA category at time of waitlist registration: 0%. 1%–84%. 85%–95%. and 96%–100%. Values are presented as number of patients (percentage within each cPRA category). Glomerular disease was the most common diagnosis across all strata. The proportion of patients with chronic tubulointerstitial nephropathy or uropathy increased with higher sensitization. whereas diabetic nephropathy decreased. Other etiologies. including hereditary and vascular nephropathies. were evenly distributed.

During follow-up, 10,637 patients (73.4%) received a transplant. This rate dropped from 77.5% (cPRA of 0%) to 50.5% (≥ 96%). LDKT was uncommon overall (10.4%) and lowest among cPRA ≥ 96% (5.8%). Mean time to transplant increased with sensitization (1.59–2.71 years). Accordingly, the proportion still on the list at last follow-up increased from 11.1% to 27.2%. Death occurred in 8.0% overall, increasing to 14.9% in cPRA ≥ 96%. Delisting was rare (4.4%) and similar across groups.

### Competing Risk Analysis: Access to Transplantation

Patients were stratified into 12 groups according to their cPRA at listing as follows: 0%: 7937 patients (54.8%); 1% to 25%: 1936 patients (13.4%); 26% to 50%: 1308 patients (9.0%); 51% to 84%: 1441 patients (10.0%); 85% to 89%: 310 patients (2.1%); 90% to 94%: 394 patients (2.7%); 95%: 99 patients (0.7%) ; 96%: 121 patients (0.8%); 97%: 149 patients (1.0%); 98%: 190 patients (1.3%); 99%: 492 patients (3.4%); and 100%: 108 patients (0.7%).

**Figure 1:**
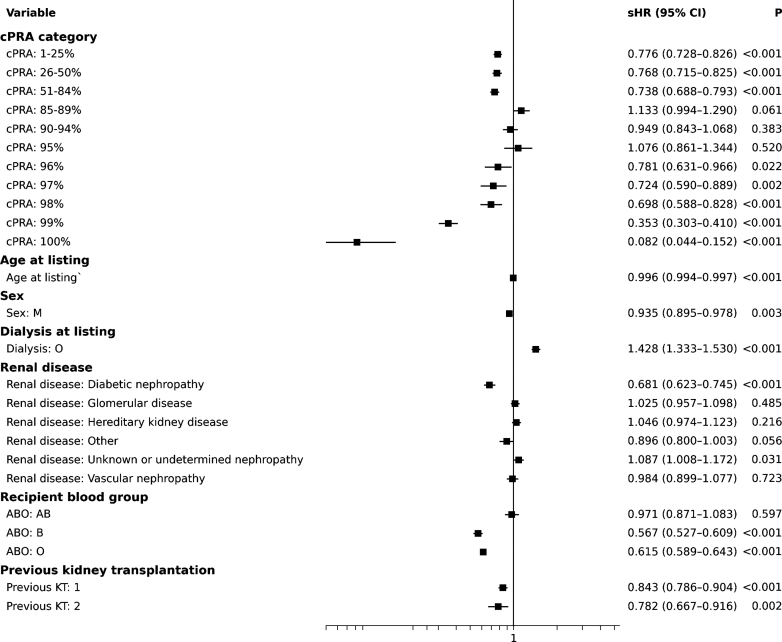
Factors associated with access to deceased-donor kidney transplantation. Forest plot of sHRs with 95% CIs for access to deceased-donor kidney transplantation, estimated using Fine and Gray competing-risk regression. Death or delisting for medical deterioration and living-donor kidney transplantation were treated as competing events. Estimates were adjusted for age at listing, gender, dialysis status at listing, recipient blood group, primary renal disease, number of previous kidney transplantations, and sensitization level (cPRA categories; reference = 0%). Analyses were limited to a maximum follow-up of 10 years. The vertical line indicates the null value (sHR = 1). CI, confidence interval; cPRA, calculated panel reactive antibody; KT, kidney transplantation; M, male; sHR, subdistribution hazard ratio.

In Fine and Gray models (Figure 1), several factors were associated with reduced transplant access:

- Older age, male gender, blood group B or O, and dialysis at listing were all associated with lower access.
- Previous transplants and diabetic nephropathy also reduced access significantly.

Immunologic sensitization showed a progressive decline in access across the cPRA spectrum. Interestingly, candidates with cPRA of 85% to 95% had similar or even slightly better access than some in the 1% to 84% range (e.g., 85%–89%: sHR = 1.133; 90%–94%: sHR = 0.949). In contrast, transplant probability was markedly lower for cPRA ≥ 99% (99%: sHR = 0.353; 100%: sHR = 0.082, both *P* < 0.0001).

### Cumulative Incidence of DDKT

**Figure 2:**
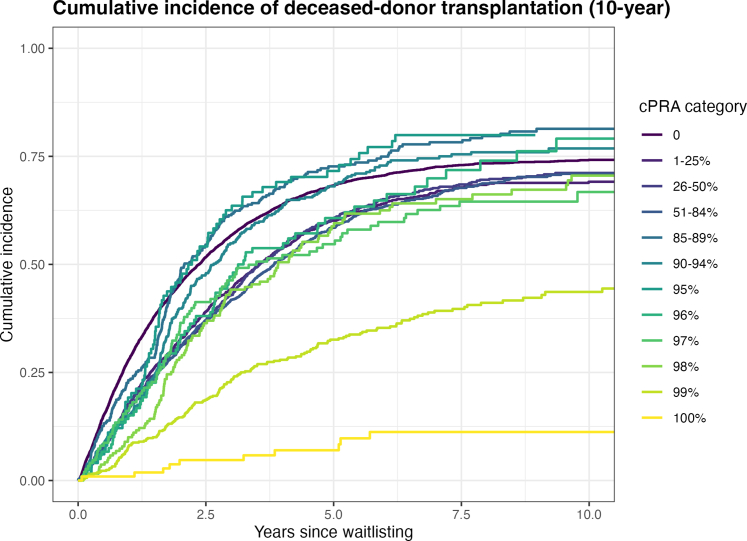
Cumulative incidence of deceased-donor kidney transplantation by sensitization level. Cumulative incidence functions for deceased-donor kidney transplantation according to cPRA categories. Death or delisting for medical deterioration and living-donor kidney transplantation were treated as competing events. Curves are truncated at 10 years after waitlisting. cPRA, calculated panel reactive antibody.

In Figure 2, we show transplant cumulative incidence stratified by cPRA. Curves diverged early and remained separated. LDKT, delisting for medical deterioration, and death were treated as competing risks. At 10 years, cPRA of 0%: 74.1%, 1% to 25%: 69%, 26% to 50%: 71% , 51% to 84%: 70.8%, 85% to 89%: 81.1%, 90% to 94%: 76.4%, 95%: 79.9% , 96%: 77.6%, 97%: 65.6% , 98%: 70%, 99%: 43.3%, and 100%: 10.5%.

### Competing Risk Analysis: Death or Delisting

**Figure 3:**
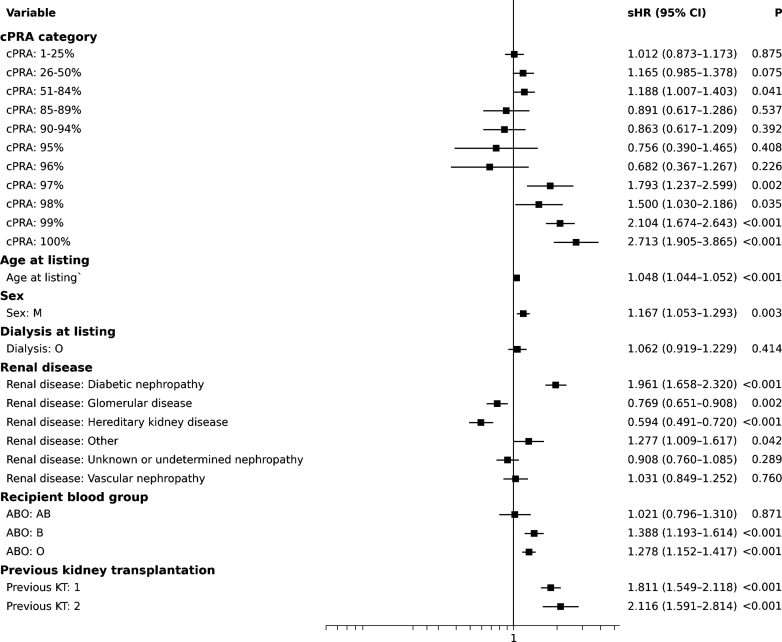
Factors associated with waitlist attrition before transplantation. Forest plot of sHRs with 95% CIs for waitlist attrition, defined as death or delisting for medical deterioration prior to any transplantation. Deceased-donor and living-donor kidney transplantation were treated as competing events. Analyses were restricted to pretransplant outcomes and estimated using Fine and Gray competing-risk models, with a maximum follow-up of 10 years. The vertical line indicates the null value (sHR = 1). CI, confidence interval; cPRA, calculated panel reactive antibody; KT, kidney transplantation; M, male; sHR, subdistribution hazard ratio.

Fine and Gray competing-risks regression (Figure 3) identified several independent predictors of death or delisting for clinical deterioration, with transplantation (both deceased and living donors) treated as a competing event:

- Older age, male gender, blood group B or O, and previous transplants were associated with increased risk.
- Dialysis at listing was not significant.
- Diabetic nephropathy conferred the highest risk
- cPRA of 85% to 96% showed no excess risk compared to cPRA 0%.

In contrast, risk increased sharply from cPRA ≥ 97%: sHR = 97%: 1.79 (*P* = 0.002), 98%: 1.50 (*P* = 0.035), 99%: 2.10 (*P* < 0.0001) and 100%: 2.71 (*P* < 0.0001).

### Cumulative Incidence of Death or Delisting

**Figure 4:**
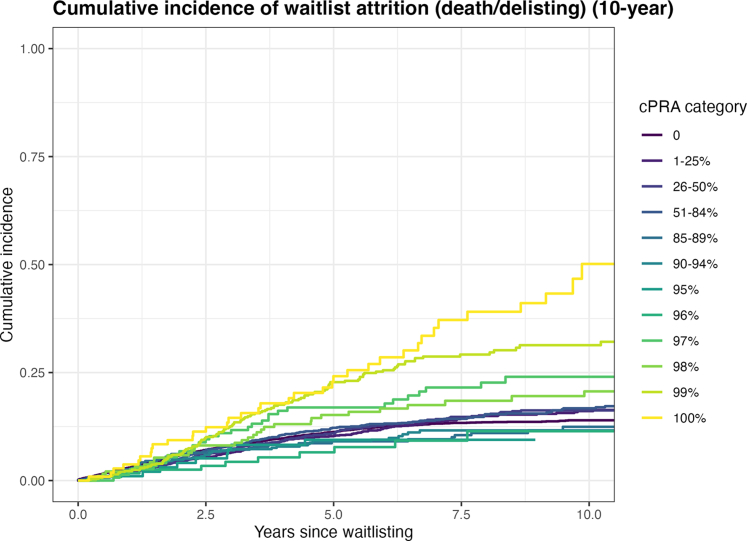
Cumulative incidence of waitlist attrition by sensitization level. Cumulative incidence of waitlist attrition (death or delisting for medical deterioration) according to cPRA categories. Deceased-donor and living-donor kidney transplantation were treated as competing events. Estimates are shown up to 10 years after waitlisting. cPRA, calculated panel reactive antibody.

In Figure 4, we display Fine and Gray cumulative incidence curves for the composite outcome of death or removal from the waiting list due to clinical deterioration, with kidney transplantation treated as a competing event. Cumulative incidence curves remained close until year 3, then diverged. At 10 years: cPRA of 0%: 13.9%; 85% to 96%: generally < 16%; 97%: 24.0%; 98%: 19.6%; 99%: 31.3%; and 100%: 46.7%.

This reveals a nonlinear risk pattern, with marked deterioration beyond cPRA 97%.

### Proportional Hazards Assumption

We assessed the proportional hazards assumption using Cox models. Schoenfeld residuals revealed a global violation (*P* < 0.0001), especially for cPRA, ABO, dialysis, renal disease, and living donor status. In contrast, age, gender, and prior transplants met proportionality.

Considering that Fine and Gray models are not constrained by this assumption, our results remain valid. Spline modeling further addressed potential time-varying effects.

## Discussion

In this national cohort, access to DDKT declined progressively with higher sensitization, with a marked decrease from cPRA ≥ 96%, while waitlist death or delisting increased from cPRA ≥ 97%. By contrast, candidates with cPRA of 85% to 95% showed transplant probabilities comparable to 1% to 84%, albeit lower than unsensitized candidates—an effect consistent with the intended impact of the French AM program in that range. These dual thresholds delineate a subset of ultrasensitized candidates (≥ 96 to 97%) who remain disadvantaged despite prioritization and should be considered for additional, targeted measures.

These observations align with, and extend, previous literature from Europe and North America. In a large US registry analysis spanning multiple allocation eras, Sapir-Pichhadze *et al.* reported a stepwise increase in waitlist mortality with rising PRA, independent of comorbidity burden and even after accounting for competing risks, underscoring the biological and logistical burden imposed by sensitization.^5^ Across Europe, several analyses have documented similar disadvantages at the highest immunologic strata, with persistent inequity at the extreme end despite prioritization tools.^15^^,^^16^

Within Eurotransplant, Ferrante *et al.*^13^ quantified transplant probability across sensitization strata using cumulative-incidence approaches, showing progressive curve flattening at very high sensitization levels. Importantly, within the Eurotransplant AM program, offer probability and realized transplantation vary even above the AM entry threshold, indicating that targeted pathways may preserve access up to a point but still leave residual constraints at the extreme tail.^17^ North American experience after allocation reform is directionally consistent. The US Kidney Allocation System introduced a cPRA-based prioritization scheme that confers stepwise benefits from ≥ 98% upward. Postimplementation evaluations^18^ documented substantial gains in access for cPRA of 98% to 99.9%, but persistent undertransplantation for ≥ 99.9% (adjusted incidence rate ratios ≈ 0.29–0.56).^19^ Centralized initiatives such as PATHI, in Spain, reported very low transplantation at cPRA of 100% despite good short-term outcomes when transplantation occurred.^20^ Collectively, these data support a consistent message: policy-based prioritization improves access for many sensitized candidates; however, inequity persists for the most highly sensitized—mirroring the ≥ 96% to 97% thresholds observed here.

Interpreting our results requires system-specific context. In France, sensitization is measured with the TGI, an integer percentage (0%–100%) representing the share of ABO-identical deceased donors from the previous 5 years against whom a candidate has ≥ 1 unacceptable anti-HLA specificity (HLA-A, -B, -DR, -DQ) recorded in CRISTAL; the metric is recalculated nightly and underpins AM eligibility at ≥ 85%, with within-AM ranking primarily driven by TGI. All analyses were performed using TGI; cPRA strata are used as internationally recognizable labels with numerically identical integer values, without implying equivalence of underlying reference populations or computational methods. This matters for interpretation: US cPRA is frequency-table–based with decimal granularity and a 2023 revision, whereas Eurotransplant defines AMs at allelic or split levels—so thresholds and prioritization effects are embedded in system design and cannot be directly extrapolated to France. Our data indicate that cPRA of 85% to 95% likely reflects a successful, by design, AM effect: access in this group is preserved or slightly better than in 1% to 84%, consistent with conferring targeted advantage just above the eligibility threshold.^21^^,^^22^ In contrast, inequity emerges from ≥ 96% to 97%, which is directly supported by our competing-risk estimates and cumulative-incidence patterns in the French TGI-based system.

Linking these empirical findings to policy, our data directly support the need for refined prioritization above the current “high sensitization” label, specifically within the 96% to 100% range, because access deteriorates sharply from ≥ 96% whereas attrition increases from ≥ 97%. The allocation options discussed below are therefore framed as system-adapted levers: they are informed by international experience (e.g., tiered Kidney Allocation System prioritization, Eurotransplant AM practices, centralized programs such as PATHI) but require evaluation within the constraints of the French TGI-based framework. To effectively target extreme compatibility scarcity, several complementary allocation levers can be considered, beyond the existing French acceptable-antigen pathway (currently activated for candidates with TGI ≥ 85% after ∼ 18 months on the waitlist). First, nonlinear prioritization tiers within the 96% to 100% range could be implemented, with progressively larger priority increments at 96%, 97%, 98%, 99%, and 100%; such microstratification would counterbalance the intrinsic limitations of an integer-based TGI and better approximate the exponential decline in compatible donors at the extreme end of sensitization. Second, a “rare-match first-offer” rule could be activated when a donor is compatible with only a very small number of candidates nationwide, ensuring that ultrasensitized recipients are not passed over in situations of exceptional immunologic opportunity. Third, optimizing the AM pathway through granular acceptable-antigen curation, including, when immunologically justified, Cw, DR51/52/53 and DP typing, or selectively incorporating allelic-level AMs after expert review, may safely expand the pool of accessible donors, drawing on principles already embedded in Eurotransplant practice. In addition, policy refinements specific to the French pathway can be considered. Given that AM activation for TGI ≥ 85% currently requires approximately 18 months of listing, shortening or waiving this waiting-time criterion for candidates with TGI ≥ 96% could mitigate early attrition in the ultrasensitized. Conversely, raising the TGI threshold for AM entry could unintentionally disadvantage candidates in the 85% to 95% range, where access appears preserved and consistent with the intended AM effect; so we do not advocate increasing the entry threshold; rather, we favor earlier activation and added priority for the most highly sensitized. In line with this, additional points for TGI ≥ 96%—analogous to the US Kidney Allocation System step-ups at very high cPRA—can be considered to create a clear, nonlinear gradient of priority at the top end. Finally, broader use and optimization of living-donor strategies—including expansion of the French kidney paired donation program, which currently remains limited compared with large-scale exchanges—may represent an important complementary pathway for ultrasensitized candidates. Collectively, these levers are intended to complement the existing acceptable-antigen framework while targeting the specific failure zone identified by our data (≥ 96% to 97%).

When optimized allocation proves insufficient—particularly ≥ 96% to 97%—a stepwise, individualized approach is needed. In selected cohorts with cPRA ≥ 99.9%, prospective multicenter data^23^ indicate that structured antibody profiling and proactive list management (antibodies delisting) can unlock transplantation in candidates previously deemed ineligible, emphasizing the value of case-by-case escalation once allocation has been exhausted. Desensitization remains a viable option but carries nontrivial risks, notably antibody-mediated rejection and infectious complications under intensified immunosuppression.^24^, ^25^, ^26^ These risks must be weighed against the well-documented survival and quality-of-life benefits of transplantation over dialysis.^10^^,^^27^ Among emerging tools, imlifidase transiently cleaves IgG and can enable transplantation across a positive crossmatch in selected patients.^28^ In France, consensus guidance from the French Society of Transplantation^29^ proposes structured selection criteria (e.g., very high sensitization such as cPRA ≥ 98%, age ≤ 65 years, > 3 years on the waitlist), donor requirements, and intensive posttransplant monitoring, offering a standardized framework for carefully selected desensitization.

This study has notable strengths. It leverages a large national cohort with 10-year observation, comprehensive immunologic characterization, and appropriate competing-risk methods to account for transplantation as a competing event; the long window enhances both robustness and temporal generalizability. To improve readability for non-French audiences, we use broader cPRA strata to describe baseline characteristics (ensuring adequate cell sizes and interpretability), and figures or analyses apply finer categories to capture high-end gradients—an approach described in the Methods. Limitations of this study include lack of epitope-level data and the fact that, although TGI is recalculated nightly, we modelled the most recent TGI value, because full time-varying reconstruction was not feasible; this choice reflects how TGI is operationally used in daily allocation while acknowledging a potential source of residual confounding. Comorbidities such as diabetes and cardiovascular disease were not captured systematically; inclusion of primary kidney disease (e.g., diabetic nephropathy) partially addresses this risk profile. This choice reflects the primary objective of evaluating allocation-related inequities rather than constructing a prognostic model of mortality, for which broader comorbidity adjustment would be required. Finally, although proportional-hazards assumptions were imperfect for some covariates, subdistribution-hazard estimates remain valid in our competing-risk framework; and sensitivity analyses yielded results consistent with the main findings.

In summary, we identify actionable thresholds—cPRA ≥ 96% for impaired access and ≥ 97% for adverse waitlist outcomes—within a TGI-based system that currently lacks graduated prioritization tiers > 85%. The apparent success of AM in 85% to 95% contrasts with persistent disadvantage of ≥ 96% to 97%, thereby arguing for targeted policy refinement and individualized pathways. Whereas our findings directly establish the French “failure zone” (≥ 96% to 97%), the proposed levers are deliberately presented as options informed by international experience that require system-specific assessment in France.

## Disclosure

All the authors declared no competing interests.
